# Preclinical assessment of IRDye800CW‐labeled gastrin‐releasing peptide receptor‐targeting peptide for near infrared‐II imaging of brain malignancies

**DOI:** 10.1002/btm2.10532

**Published:** 2023-05-09

**Authors:** Yuan Zhang, Li Wang, Chengkai Zhang, Jingjing Zhang, Linhao Yuan, Shucheng Jin, Wenjianlong Zhou, Xiudong Guan, Peng Kang, Chuanbao Zhang, Jie Tian, Xiaoyuan Chen, Deling Li, Wang Jia

**Affiliations:** ^1^ Department of Neurosurgery, Beijing Tiantan Hospital Capital Medical University Beijing China; ^2^ Beijing Neurosurgical Institute Beijing China; ^3^ Jiangsu Xinrui Pharmaceutical Co., Ltd. Nantong China; ^4^ Departments of Diagnostic Radiology, Surgery, Chemical and Biomolecular Engineering, and Biomedical Engineering, Yong Loo Lin School of Medicine and Faculty of Engineering National University of Singapore Singapore Singapore; ^5^ Clinical Imaging Research Centre, Centre for Translational Medicine, Yong Loo Lin School of Medicine National University of Singapore Singapore Singapore; ^6^ Nanomedicine Translational Research Program, NUS Center for Nanomedicine, Yong Loo Lin School of Medicine National University of Singapore Singapore Singapore; ^7^ CAS Key Laboratory of Molecular Imaging, Beijing Key Laboratory of Molecular Imaging, The State Key Laboratory of Management and Control for Complex Systems Institute of Automation, Chinese Academy of Sciences Beijing China; ^8^ School of Artificial Intelligence University of Chinese Academy of Sciences Beijing China; ^9^ Beijing Advanced Innovation Center for Big Data‐Based Precision Medicine, School of Medicine Beihang University Beijing China

**Keywords:** brain malignancies, fluorescence guided surgery, GRPR, NIR‐II imaging

## Abstract

We aimed to develop a new biocompatible gastrin‐releasing peptide receptor (GRPR) targeted optical probe, IRDye800‐RM26, for fluorescence image‐guided surgery (FGS) of brain malignancies in near‐infrared window II (NIR‐II) imaging. We developed a novel GRPR targeting probe using a nine‐amino‐acid bombesin antagonist analog RM26 combined with IRDye800CW, and explored the fluorescent probe according to optical properties. Fluorescence imaging characterization in NIR‐I/II region was performed in vitro and in vivo. Following simulated NIR‐II image‐guided surgery, we obtained time‐fluorescent intensity curves and time‐signal and background ratio curves. Further, we used histological sections of brain from tumor‐beating mice model to compare imaging specificity between 5‐aminolevulinic acid (5‐ALA) and IRDye800‐RM26, and evaluated biodistribution and biocompatibility. IRDye800‐RM26 had broad emission ranging from 800 to 1200 nm, showing considerable fluorescent intensity in NIR‐II region. High‐resolution NIR‐II imaging of IRDye800‐RM26 can enhance the advantages of NIR‐I imaging. Dynamic and real time fluorescence imaging in NIR‐II region showed that the probe can be used to treat brain malignancies in mice between 12 and 24 h post injection. Its specificity in targeting glioblastoma was superior to 5‐ALA. Biodistribution analysis indicated IRDye800‐RM26 excretion in the kidney and liver. Histological and blood test analyses did not reveal acute severe toxicities in mice treated with effective dose (40 μg) of the probe for NIR‐II imaging. Because of the considerable fluorescent intensity in NIR‐II region and high spatial resolution, biocompatible and excretable IRDye800‐RM26 holds great potentials for FGS, and is essential for translation into human use.

**Abbreviations**
5‐ALA: 5‐aminolevulinic acid
FGS: fluorescence guided surgery
GRPR: gastrin‐releasing peptide receptor
ICG: indocyanine green
MFI: mean fluorescence intensities
NIR: near infrared
PBS: phosphate buffer saline
SBR: signal and background ratio

## 1 INTRODUCTION

Brain malignancies such as glioblastomas and brain metastases are the most aggressive brain tumors.^1^ The extent of resection is a crucial factor for patient prognosis in neurosurgery.^2^, ^3^ However, brain malignancies have traditionally been considered poorly delineated and invasive,^4^ thereby making safe maximal resection by naked eye tough.

Fluorescence image‐guided surgery (FGS) provides real‐time image guidance and improves intraoperative visualization of brain tumor, independent of brain shift than neuronavigation.^5^ Preclinical and clinical studies of FGS using the Food and Drug Administration (FDA) approved fluorescent probes fluorescein,^6^ 5‐aminolevulinic acid (5‐ALA),^7^ indocyanine green (ICG),^8^, ^9^ and methylene blue^10^ showed preliminary successes. However, their applications are limited by insufficient accumulation in tumors, high tissue autofluorescence, or poor tissue penetration.

Because the fluorescence imaging can be improved by extending the wavelength beyond 1000 nm, fluorescence imaging in the near infrared II (NIR‐II) (1000–1700 nm) region is more favorable, providing a high platform for in vivo bioimaging to burrow deeper into the tissues with a clearer observation.^11^, ^12^ However, for clinical translation, the existing NIR‐II fluorescent probes, including many molecular dyes and inorganic nanomaterials raised concerns in terms of their complicated synthesis routes and unknown immunogenic responses.^13^ Due to the absence of FDA‐approved probes with peak emission in the NIR‐II region, molecular imaging investigations of NIR‐I cyanine dyes, including ICG, IRDye800CW, and IR‐12N3 revealed long emission tails that stretch into the NIR‐II region, developing a new strategy for clinical NIR‐II fluorescence imaging.^14^, ^15^, ^16^, ^17^, ^18^

High levels of gastrin‐releasing peptide receptor (GRPR) expression have been observed in many tumor types^19^ and have been connected with epithelial‐to‐mesenchymal transition and cell proliferation, migration, invasion, and survival,^20^, ^21^, ^22^ indicating its potential for diagnosis and treatment. Although we previously exploited probes that proved benefits of an agonist peptide (Bombesin) for FGS with IRDye800CW and made a good performance to visualize glioblastoma within the NIR‐I window (700–900 nm),^23^, ^24^, ^25^ further improvements and optimization may be imperative such as obtaining clearer visualization within the NIR‐II window.

IRDye800CW and RM26 (a nine‐amino‐acid bombesin antagonist analog D‐Phe‐Gln‐Trp‐Ala‐Val‐Gly‐His‐Sta‐Leu‐NH2) have made great achievements in tumor‐targeting imaging^26^, ^27^; we therefore developed and proofed a novel GRPR targeting probe using RM26 in combination with the IRDye800CW fluorophore for NIR‐II imaging guided neurosurgery. In this study, the remarkable signal and background ratio (SBR) was capable of completely removing glioblastoma with tumor‐bearing mice under NIR‐II imaging navigation. Interestingly, IRDye800‐RM26 was more effective than 5‐ALA for glioblastoma targeting. Furthermore, the NIR‐II fluorescent signal accumulation in most organs and blood dropped approximately 89%–99% in 24 h after intravenous administration. Overall, this study demonstrated that IRDye800‐RM26 with outstanding performance in NIR‐II imaging properties, excretability, and biocompatibility is suitable for use in fluorescence‐guided neurosurgery preclinically and facilitate its future clinical translation.

## 2 MATERIALS AND METHODS

All experimental procedures in animals were approved by the Animal Care and Use Committee of Beijing Neurosurgical Institute and performed in accordance with the ARRIVE (Animal Research: Reporting in vivo Experiments) guidelines.

### 2.1 Synthesis of GRPR‐targeted imaging agents

**FIGURE 1:**
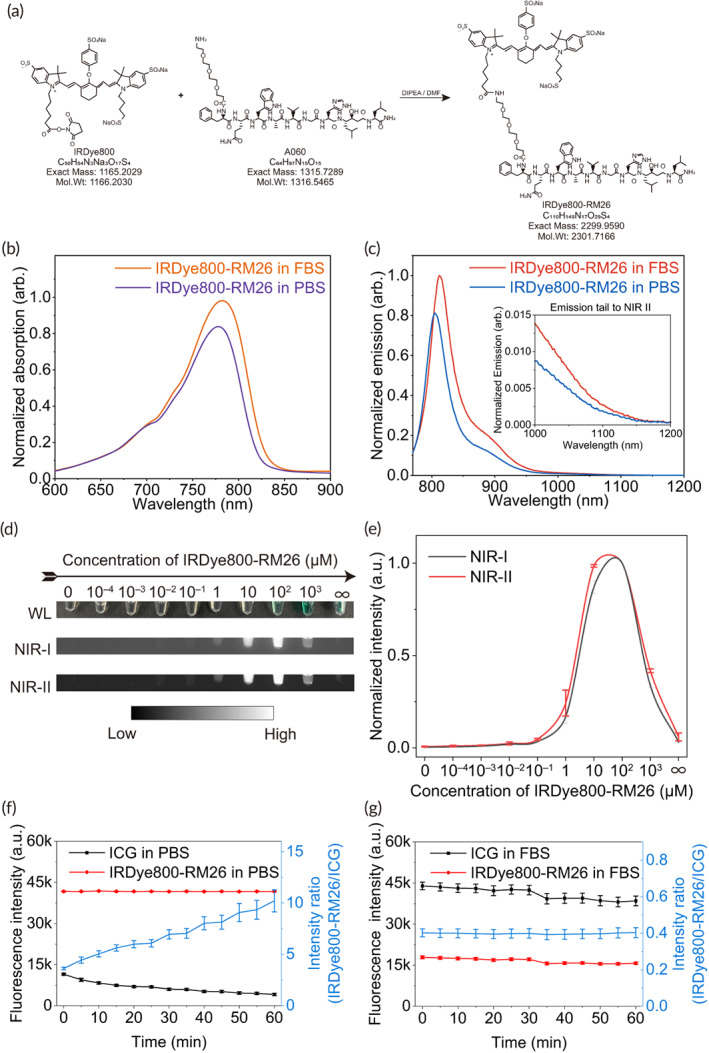
Optical property. (a) Synthetic process and molecular structure of IRDye800‐RM26. (b and c) Spectral analysis of the probe with absorption peak at 777 nm in PBS and 783 nm in FBS; and emission (758 nm excitation) peak at 805 nm in PBS, and 813 nm in FBS; and tail emission of IRDye800‐RM26 in the 1000–1200 nm range. (d and e) NIR‐II fluorescence imaging and mean fluorescence intensity of different concentrations of IRDye800‐RM26. (f and g) Changes in fluorescence intensity of IRDye800‐RM26 and ICG in PBS and FBS continuous exposed to 808 nm light for 1 h.

To a 25 mL glass vial containing 90 mg RM26 in 12 mL of DMF 80 mg of IRdye800CW NHS ester (LI‐COR, Lincoln, Nebraska) and 90 μL DIPEA were added. The mixture was stirred at room temperature for 1 h and purified with HPLC to give 15 mg of the desired product with 10% yield and >95% purity. LC–MS: [(MHH)/2] ++ = 2299.9590 (m/z), calc: 2301.7166 (C_110_H_149_N_17_O_29_S_4_) (Figure 1).

### 2.2 Fluorescence imaging in NIR‐I and NIR‐II window

We acquired NIR images using the Medical camera Cheetah series (Xenics, Singapore) for NIR‐II imaging through a 1000 nm long pass filter and pco.edge 5.5 (PCO, Germany) for NIR‐I imaging.

To compare the penetration of the probe in NIR‐I and NIR‐II windows, a fresh mouse brain was cut into 1 mm to 4 mm slices with brain matrices (RWD, China) to cover a capillary glass tube (inside diameter = 0.3 mm) filed with 100 μM IRDye800‐RM26 in fetal bovine serum (FBS). The tube was excited by an 808 nm laser at 500 ms exposure time using power density of approximately 1.3 mW/cm^2^ of light, below the limit of 329 mW/cm^2^ established for safe exposure. A full‐width‐half‐maximum (FWHM) analysis was performed at different depths in the two windows.

To compare dosage‐dependent contrast of images in NIR‐I and NIR‐II windows, we diluted IRDye800‐RM26 to concentrations of 10 μM (≈4 μg of the probe), 100 μM (≈40 μg of the probe), and 1000 μM (≈400 μg of the probe) in 1 × PBS and injected them into the tail vain of each tumor‐bearing (GL261 cells) C57BL/6 mouse (*n* = 3). After 6 h, the mice were euthanized with overdose of 2,2,2‐tribromoethanol. The brains were removed and excited with diffuse 792 nm excitation (12.9–65.8 mW/cm^2^) at 500 ms exposure time for NIR‐I and NIR‐II imaging. We quantified the contrast within region of interests (ROIs) containing the tumor boundaries in both NIR‐I and NIR‐II imaging by calculating the signal intensity, coefficient of variation (defined as the standard deviation of pixel intensity normalized to the mean pixel intensity) and SBR.

### 2.3 The time window of NIR‐II imaging for brain tumors

Tumor‐bearing female mice (*n* = 1 mouse was in per timepoint) were euthanized after 1, 3, 6, 12, and 24 h post injection of 40 μg of the probe for ex vivo dynamic NIR‐II imaging. A tumor‐bearing mouse was not injected and served as control. The SBR was calculated. Tumor signal was measured as a mean fluorescence intensity (MFI) of tumor within the ROIs containing the tumor boundaries, and background signal was measured as the MFI of a representative area with no tumor within ROIs containing the tumor boundaries. Subsequently, we removed tumors with high fluorescent intensities (guided by NIR‐II fluorescence of IRDye800‐RM26) and peritumoral brain tissues with low fluorescent intensities. Following NIR‐II imaging guided surgical simulation, the tumor and peritumor histology and morphology was observed with H&E stain, and imaged them with automated quantitative pathology imaging system (Vectra Polaris 1.0.7, Akoya Bioscience).

In vivo dynamic NIR‐II imaging of IRDye800‐RM26 through a cranial window in nude female mice (*n* = 3) with brain tumor implantation using A549 cell line was implemented to reduce tumor bias (e.g., size, depth, blood supply). After 100 μM (≈40 μg of the probe) of IRDye800‐RM26 were injected into the tail vain of the mice, the mice were anesthetized and immediately imaged through cranial window using diffuse 792 nm excitation (66.3–78.7 mW/cm^2^) at 500 ms exposure time, 1000 nm long‐pass dielectric filters, and an InGaAs camera at following time points: 0.1, 0.5, 1, 2, 3, 4, 5, 6, 8, 10, 12, and 24 h. SBR were calculated using ROIs containing the tumor boundaries. Tumor signal was based on the region of tumor lesion in ROIs, and background signal was based on the corresponding region of peritumor in ROIs.

### 2.4 Targeted specificity with IRDye800‐RM26

We incubated A549, MDA‐MB‐231, and GL261 cells with 100 μM IRDye800‐RM26 for 1 h. Subsequently, we washed the cells with 1 × PBS 3 times and scanned then using fluorescence microscopy (BZ‐X800; KEYENCE, Japan). The NIR emission was collected with an excitation filter (775 ± 50 nm), dichromatic mirror (810 nm), and a barrier filter (845 ± 55 nm). The blocking dose of 500 μM RM26 was administered before cells were incubated with 100 μM IRDye800‐RM26 and imaged after 1 h.

We equally analyzed the histological colocalization of the fluorescence signal and cancer cells. The mouse was euthanized and the brain was removed at 3 h after a tumor‐bearing (A549 cells) nude female mouse was injected with 100 μM (≈40 μg of the probe) IRDye800‐RM26. Frozen sectioning was performed after fixation of the brain specimen in Tissue‐Tek O.C.T. using a cryostat. The fixed tissue was sliced axially at 30 μm thickness and mounted on a slide for immediate fluorescence micro imaging with the BZ‐X800. Multiple slices were performed across the tumor. Subsequently, we stained some slices with H&E according to common standard procedure, and imaged them with BZ‐X800 or Vectra Polaris. In addition, 120 mg/kg of 5‐ALA (Sigma‐Aldrich) was injected intraperitoneally into a tumor‐bearing (GL261 cells) C57BL/6 female mouse, and then 100 μM (≈40 μg of the probe) IRDye800‐RM26 was injected intravenously. The mouse was euthanized and prepared for section imaging at 4 h post‐injection. The 5‐ALA emission was collected with an excitation filter (545 ± 25 nm), dichromatic mirror (565 nm), and a barrier filter (605 ± 70 nm). Quantification of the colocalization of tumor on H&E staining, 5‐ALA, and NIR were performed with the JACoP plugin for ImageJ using the Manders' coefficients.^28^ Values of these coefficients range from 0 to 1 and express the fraction of intensity in a channel located in pixels where there is above threshold intensity in the other color channel.

### 2.5 Biodistribution and excretion of IRDye800‐RM26

The C57BL/6 female mice (*n* = 1 mouse was in per timepoint) were intravenously injected with 100 μM (≈40 μg of the probe) IRDye800‐RM26. Blood was collected from the orbit, and organs (heart, liver, spleen, lung, kidney, brain) were removed at various time points post injection (0, 1, 3, 6, 12, 24 h). Then dynamic NIR‐II fluorescence images of blood and organs were recorded with the 793 nm laser (about 27.9 mW/cm^2^).

### 2.6 Biocompatibility of IRDye800‐RM26

We measured blood routine indexes in C57BL/6 female mice receiving 100 μM (≈40 μg of the probe) IRDye800‐RM26 before and 3 days after. We measured the blood biochemical parameters before and 3, 7, and 14 days after administration. All assays were repeated three times. Besides, the mice were euthanized, and vital organs (heart, liver, spleen, lung, kidney, brain, pancreas, and digestive tract) were harvested before and 3 days after injection of the probe at the same doses. The samples were embedded in paraffin, sectioned into 4 μm slices, stained with H&E, and imaged them with Vectra Polaris.

### 2.7 Statistics

Graphing, data analysis, and statistics were performed in OriginPro 2021 version 9.8.0.200 (OriginLab) using the unpaired Student's *t*‐test unless otherwise stated.

## 3 RESULTS

### 3.1 Optical properties in NIR‐II region

The visible and NIR emission spectra demonstrated that the emission of IRDye800‐RM26 in PBS extended into the NIR‐II region (>1000 nm) under 758 nm excitation, even though the emission peak was 805 nm (Figure 1b,c). In addition, IRDye800‐RM26 became brighter in fetal bovine serum (FBS), and its emission peak presented a Stokes shift of 8 nm when excited with 758 nm laser. IRDye800‐RM26 exhibited concentration‐dependent fluorescence quenching (Figure 1d,e). The highest NIR‐II fluorescent intensity in FBS was observed at the concentration of 100 μM. The comparison of the brightness between IRDye800‐RM26 and ICG showed that the brightness of IRDye 800‐RM26 were 3.6 and 0.4 times, respectively, that of ICG in PBS and FBS when excited with 808 nm light in NIR‐II window. However, after continuous exposed to 808 nm light for 1 h, the brightness of IRDye800‐RM26 were 10.2 and 0.4 times, respectively, that of ICG in PBS and FBS (Figures 1f,g and S1). These results indicated that IRDye800‐RM26 exhibited higher brightness in PBS, but lower brightness in FBS compared with ICG. Besides, the photostability of IRDye800‐RM26 in PBS was better than ICG, and IRDye800‐RM26 also exhibited excellent photostability in FBS.

### 3.2 NIR‐II fluorescence imaging outperformed NIR‐I

**FIGURE 2:**
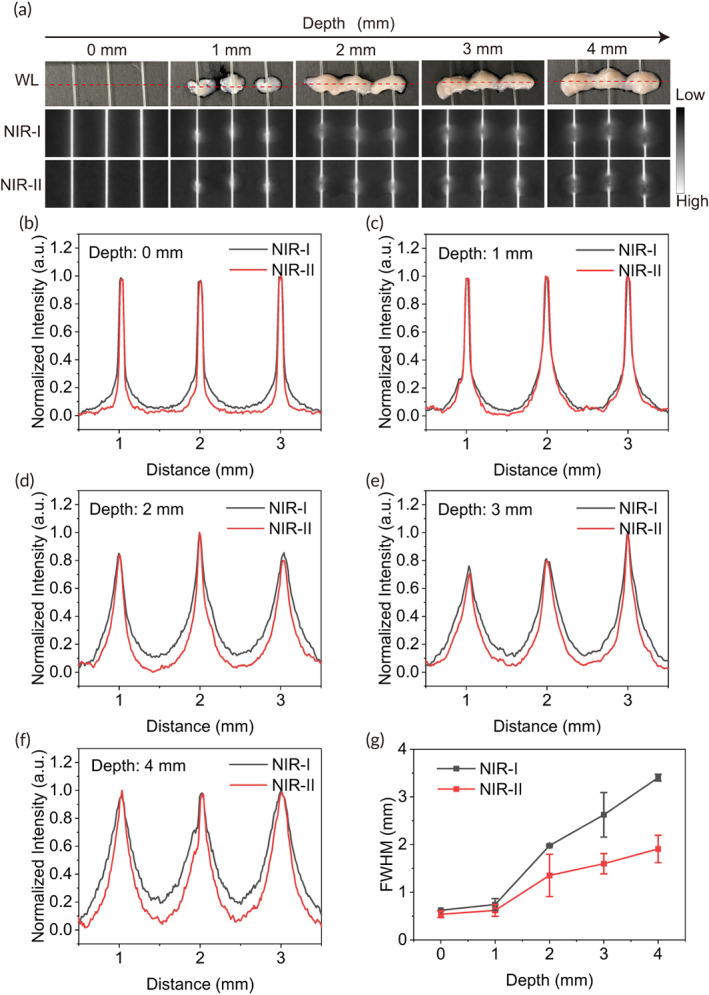
NIR‐I/II penetration depth of IRDye800‐RM26. (a) NIR‐I/II fluorescence images, and (b–f) cross‐sectional fluorescence intensity profiles along red‐dashed bars of glass capillary tubes with the IRDye800‐RM26 (100 μM). (g) Full‐width‐half‐maximum (FWHM) of capillary glass tube filled with IRDye800‐RM26 in FBS as a function of depth in brain, showing loss of feature integrity in NIR‐I compared to the NIR II.

The cross‐sectional fluorescence intensity profiles of IRDye800‐RM26 capillary images showed that at 0–4 mm the scattering effects in NIR‐I images were more pronounced than that in NIR‐II images (Figure 2a–f). The FWHM analysis of the capillary tubes at a depth of 1 mm from the brain surface measures 0.62 ± 0.12 in NIR‐II window and 0.74 ± 0.12 mm in NIR‐I window. At a depth of 4 mm from the brain surface, the tube measures 1.91 ± 0.29 mm in NIR‐II window and 3.40 ± 0.07 mm in NIR‐I window (Figure 2g).

**FIGURE 3:**
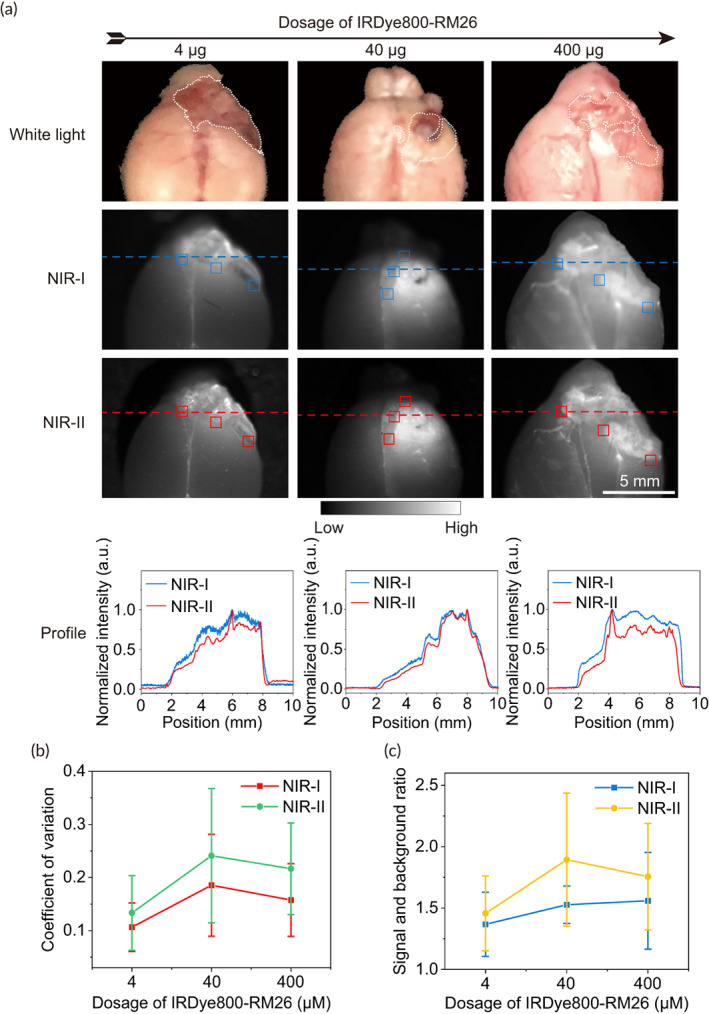
Contrast quantification of NIR‐I/II fluorescence imaging for C57BL/6 mice with glioblastoma at 6 h after tail intravenous injection of IRDye800‐RM26. (a) Ex vivo NIR‐I/II imaging and cross‐sectional intensity profile (blue dashed bars in images under NIR‐I window, or red‐dashed bars in images under NIR‐II window) of glioblastoma (white dotted lines) using different dosages of IRDye800‐RM26 at NIR‐I and NIR‐II windows. (b) The contrast, defined as the standard deviation divided by the mean pixel intensity, was quantified to compare NIR‐II and NIR‐II imaging of tumor. The mean contrasts in ROIs of tumor boundaries (blue boxes in images under NIR‐I window, or red boxes in images under NIR‐II window) were 0.106 and 0.133, 0.185 and 0.241, 0.158 and 0.217 for NIR‐I and NIR‐II imaged tumor boundaries after administration with 4, 40, and 400 μg, respectively. (c) Corresponding SBR in ROI (blue or red boxes) of NIR‐I and NIR‐II imaging for different dosages of IRDye800‐RM26.

To demonstrate potential improvement of brain tumor imaging from NIR‐I to NIR‐II window, the mouse glioblastomas were imaged with IRDye800‐RM26 in both windows (Figure 3a). Specifically, the peaks in NIR‐II cross‐sectional intensity profiles of glioblastomas were sharper than that in NIR‐I (Figure 3a, bottom). The NIR‐II images contrast for tumor boundaries is 0.133, 0.241, and 0.217 after administration with 4, 40, and 400 μg, respectively, that 20%, 23%, and 27% greater compared with the NIR‐I images (0.106, 0.185, and 0.158, respectively) (Figure 3b). Compared to the SBR in NIR‐I images, the SBR in NIR‐II images were increased by 6%, 19%, and 11%, respectively at the dosage of 4, 40, and 400 μg (Figure 3c). These findings indicated that the dramatic contrast was enhanced for NIR‐II imaging.

### 3.3 The time window of NIR‐II imaging for FGS

**FIGURE 4:**
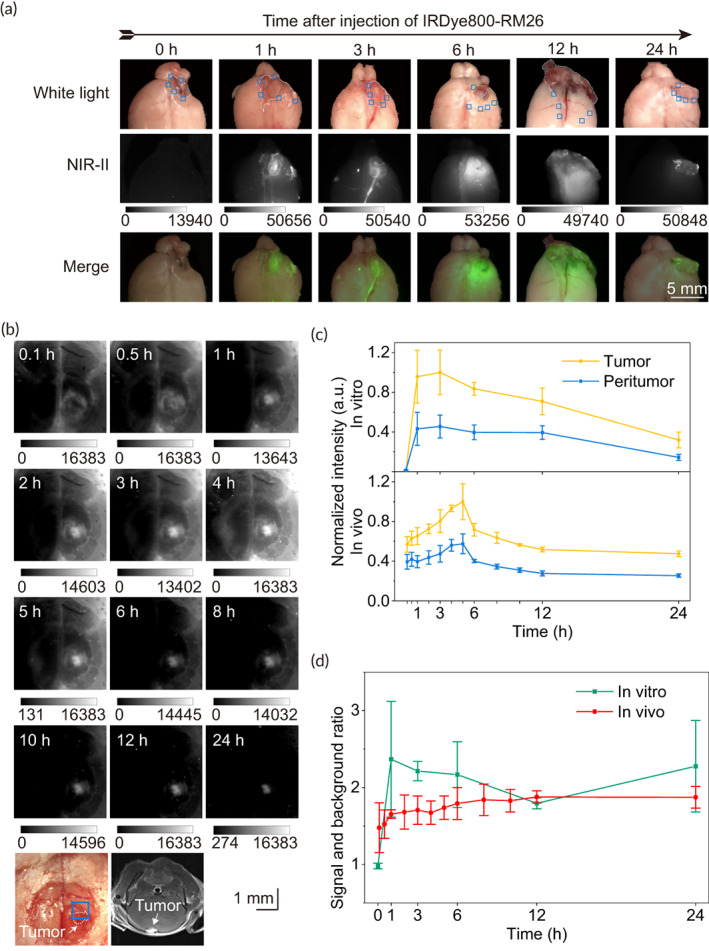
Dynamic imaging for C57BL/6 mice with glioblastoma using 40 μg IRDye800‐RM26. All data are based on images with an exposure time of 500 ms. (a) Ex vivo dynamic NIR‐II imaging of glioblastoma (white dotted lines). (b) In vivo dynamic NIR‐II fluorescence imaging through a cranial window in a nude mouse with brain tumor implantation (white arrow) using the A549 cell line. (c) Tumor and peritumor normalization fluorescence intensities in ROIs (blue boxes) of tumor boundaries under NIR‐II window ex vivo and in vivo. (d) Corresponding SBR in ROIs (blue boxes) of tumor boundaries under NIR‐II windows ex vivo and in vivo.

Inspired by the high contrast NIR‐II images of glioblastoma in vitro, we explored the time window of ex vivo and in vivo NIR‐II fluorescence imaging for surgical navigation. Ex vivo NIR‐II images revealed clear tumor visualization within 24 h in glioblastoma mouse (Figure 4a). The highest average SBR within tumor boundaries was 2.4 at 1 h after injection of a dosage of 40 μg (Figure 4d). Furthermore, the average SBR were 2.2, 2.2, 1.8, and 2.3 at 3, 6, 12, and 24 h, respectively. According to the H&E stains of the “tumor” and “peritumor” after surgery, a residual tumor was seen in the area of “peritumor” in mouse receiving surgery without the probe, while the total resection without residual tumor were achieved in the mice with simulated NIR‐II image‐guided surgery (Figure S2). In addition, compared to 12–24 h after injection, more normal brain tissues were present in “tumor” with high NIR‐II fluorescent intensities between 1 and 6 h. Therefore, during 12–24 h post intravenous administration, NIR‐II images could accurately delineate glioblastoma and help safely remove the brain tumor.

To reveal in vivo NIR‐II fluorescence imaging of brain metastasis and exclude the bias caused by individual differences in tumors, we conducted real‐time NIR‐II imaging through a cranial window in a nude mouse with brain tumor implantation using the A549 cell line (Figure 4b). The dynamic imaging of tumor in NIR‐II window presented strong signal in the tumor while very low signal was observed from brain and vessels during 12–24 h post‐injection of 40 μg IRDye800‐RM26 (Figure 4c). The SBR increased rapidly 1 h after injection, increased continuously from 1 to 6 h, and remained stable during 12–24 h (Figure 4d). Therefore, NIR‐II imaging of brain tumors presented low signals of normal brain and high SBR during 12–24 h that was the time window for FGS.

### 3.4 Tumor targeting specificity of IRDye800‐RM26

**FIGURE 5:**
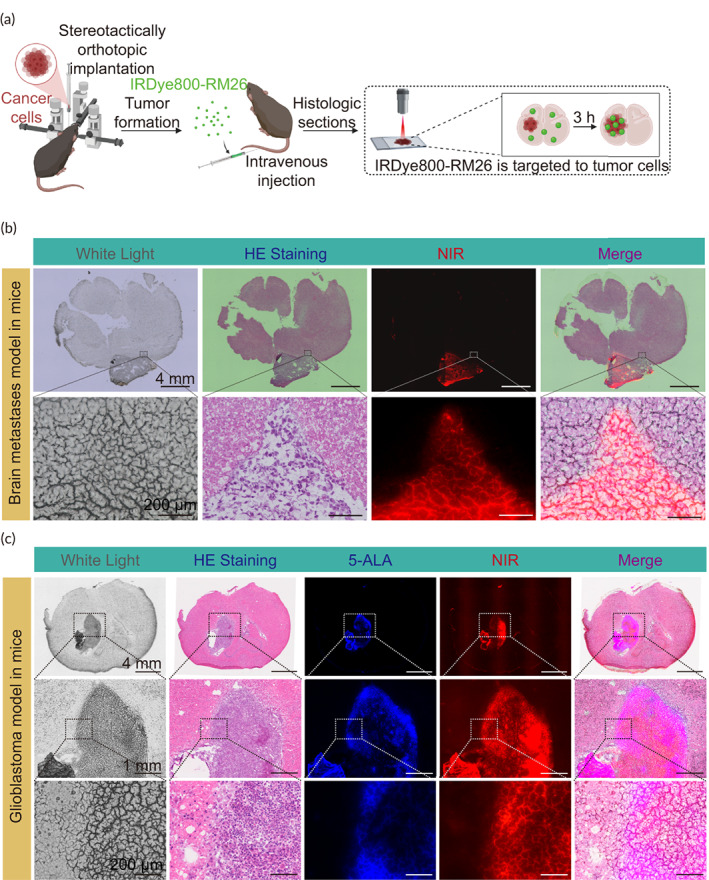
Imaging specificity of 40 μg IRDye800‐RM26. NIR can better display the interior and boundaries of the brain malignancies than white light and 5‐ALA. (a) Workflow of microscope imaging of brain malignancies (created with BioRender.com). (b) Histology of tumor (A549 cell line)‐bearing nude mouse brain with white light imaging, H&E staining, NIR micro‐imaging, and white light/H&E/NIR merged. White light images and NIR micro‐images are from the exact same slice, while the H&E staining images are from the consecutive slices. (c) Histology of tumor (GL261 cell line) bearing C57BL/6 mouse brain with white light imaging, H&E staining, 5‐ALA micro‐imaging, NIR micro‐imaging, and white light/H&E/5‐ALA/NIR merged. White light images, 5‐ALA images, and NIR micro‐images are from the exact same slice, while the H&E staining images are from the consecutive slices.

The targeted cell imaging showed successful binding between the IRDye800‐RM26 and GRPR of A549, MDA‐MB231, and GL261 cell lines (Figure S3). In addition, the brain section staining showed excellent targeting capability of the probe to GRPR (Figure 5a).

The tumor extent harvested from the mice at 4 h post‐injection of 40 μg IRDye800‐RM26 was invisible with vague demarcation from healthy tissue in white‐light high‐power field; however, it was clearly visualized in the NIR imaging (Figure 5b,c). In addition, the histological assessment of the overlap between tumor and the probe performed with Menders' coefficients. In brain metastases, 98.2% of IRDye800‐RM26 in the entire image colocalized with tumor, and 91.9% of tumor in entire image colocalized with IRDye800‐RM26. In glioblastoma, the portion of IRDye800‐RM26 that colocalized with tumor was 99.7%, and the portion of tumor that colocalized with IRDye800‐RM26 was 87.9%. However, the values of Menders' coefficients were 99.8% and 63.8%, respectively for the fraction of glioblastoma on 5‐ALA overlapping tumor on H&E, and fraction of tumor on H&E overlapping tumor on 5‐ALA. These results indicated that IRDye800‐RM26 has higher tumor targeting specificity compared to 5‐ALA.

### 3.5 Biodistribution and excretion of IRDye800‐RM26

**FIGURE 6:**
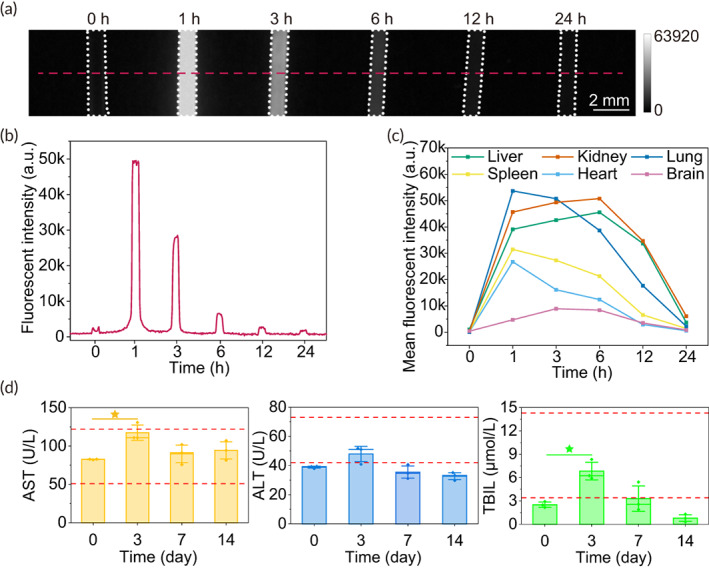
Biodistribution, excretion, and acute toxicity in C57BL/6 mice. (a and b) Ex vivo NIR‐II fluorescence imaging and cross‐sectional intensity profile (red‐dashed bars) for the blood of mouse in glass capillary tubes (white‐dotted lines) at different time after tail intravenous injection of 40 μg IRDye800‐RM26. (c) Quantification of fluorescence signal from dynamic images of organs (as shown in Figure S4) representing the biodistribution and excretion of 40 μg IRDye800‐RM26. (d) Blood chemistry profiles including AST, ALT, and TBIL. Red dash referred to 2.5th–97.5th percentiles interval of blood parameters from study by Cristina Mazzaccara et al.

To investigate the biodistribution and excretion of IRDye800‐RM26, we performed NIR‐II imaging of mouse blood and vital organs at multiple time points within 24 h post injection. The fluorescence intensity in blood was high at 1 h but extremely low at 12 h post injection (Figure 6a,b). At 1 h, due to the highest level of probe in the blood, the lung exhibited the highest MFI followed by the kidney, liver, spleen, heart, and brain, which were likely to be consistent with blood perfusion to organs. However, the kidney and liver exhibited the highest MFI followed by the lung, spleen, heart, and brain at 6 h (Figures 6c and S4). The NIR‐II fluorescence intensities dropped approximately 89% and 94% respectively in the kidney and liver at 24 h. This demonstrates that approximately 90% of IRDye800‐RM26 was excreted mainly through the kidney and liver within 24 h. Remarkably, the normal brain tissue exhibited very low NIR‐II fluorescent intensities in the whole course, beneficial to the high SBR of tumor in NIR‐II imaging.

### 3.6 Biocompatibility of IRDye800‐RM26

To evaluate the biocompatibility of IRDye800‐RM26, we conducted blood routine and biochemistry tests in C57BL/6 mice (Tables S1 and S2). Although some biomarkers were altered compared with the control groups, they maintained within 2.5th to 97.5th percentiles interval of normal.^29^, ^30^ Significantly, the level of aspartate aminotransferase approached the upper normal limit on the 3rd day, but recovered on the 7th day (Figure 6d), which assumed potential but reversible liver damage at the effective and optimal dose (40 μg). Meanwhile, the histological analysis demonstrated no distinct hydropic damage or necrotic lesions compared to the normal control groups at 3 days after tracer injection (Figure S5).

### 3.7 Preliminary studies of drug action on tumor cells

Gastrin‐releasing peptide receptor signaling contributes to the growth and metastatic potential of tumors.^21^, ^22^ After 24 h of incubation with 10, 50, and 100 μM of IRDye800‐RM26, there was no significant influence on the cell viability of A549 cells (Figure S6a). Similarly, after 24 and 48 h of incubation, IRDye800‐RM28 did not have an impact on tumor cells viability (Figure S6b). Transwell experiments showed that the number of cells penetrating the basement membrane was significantly lower in groups treated with 100 μM probe than in untreated groups in cell lines (Figure S6c,d), which was also lower to the number of cells in the group treated with 10 μM probe. Therefore, blockade of GRPR signaling using low dose of IRye800‐RM26 (10 μM) inhibited tumor cells invasion.

## 4 DISCUSSION

Near‐infrared fluorescence imaging technology is widely used for FGS due to its non‐invasive, real‐time, and multi‐dimensional monitoring characteristics. The combination of the reducing photon scattering, light absorption, and tissue autofluorescence provides a clear view deep into the body, capable of extraordinarily high SBR that are only accomplishable when moving from the NIR‐I to NIR‐II region. Because some organic dyes are relatively easy to synthesize, modifiable, and have low biological toxicity, several studies recently explored clinically approved or commercially available NIR‐I cyanine dyes (ICG, IRDye800, IR‐12N3, and IR‐820) that may serve as NIR‐II fluorophores with emission tails extending into the NIR‐II region, providing a unique opportunity to directly translate NIR‐II bioimaging into clinical theranostics.^13^, ^14^, ^17^, ^31^, ^32^ The combination of high quantum yield (QY = 12%, NIR‐I, serum) and functional groups for easy targeting moiety bioconjugation have promoted IRDye800 to the forefront of clinical near‐infrared fluorescent dyes.^11^ Shoujun Zhu and colleagues showed that IRDye800 displayed 3.3‐fold and 1.8‐fold higher brightness (NIR‐I) than ICG in PBS and FBS (100 μM), respectively.^13^ But Jessica A. Carr showed that ICG was 12.3 times brighter than IRDye800CW in bovine blood (0.01 mg/mL).^17^ The major reason of the difference was caused by the difference in concentration. Previous study suggested that the concentration of ICG with maximum fluorescence intensity was 0.01–0.02 mg/mL in BSA.^33^ In our study, the highest NIR‐II fluorescent intensity of IRDye800‐RM26 in FBS was observed at the concentration of 100 μM. Therefore, we compared the brightness between IRDye800‐RM26 (100 μM) and ICG (0.01 mg/mL), and found that the brightness of IRDye 800‐RM26 were 3.6 and 0.4 times of ICG in PBS and FBS, respectively. However, after continuous exposed to 808 nm light for 1 h, the brightness of IRDye800‐RM26 were 10.2 and 0.4 times of ICG in PBS and FBS, which indicated that IRDye800‐RM26 exhibited higher brightness in PBS, but lower brightness in FBS compared with ICG. Besides, the photostability of IRDye800‐RM26 in PBS was better than ICG, and IRDye800‐RM26 also exhibited excellent photostability in FBS. IRDye800CW has been the forefront clinical NIR fluorescent dye for image‐guided neurosurgery across NIR‐I window,^23^ but little about image‐guided neurosurgery across NIR‐II window. These properties inspired us to explore the advantages of this probe in NIR‐II imaging for FGS. In this study, we successfully synthesized a new peptide‐based fluorescent probe that effectively visualized brain malignancies. Several preclinical assessments would be used to verify that NIR‐II imaging of IRDye800‐RM26 could further enhance the advantages of NIR‐I imaging in FGS.

For NIR fluorescence imaging in FGS, the timing of uptake of the probes would be highly convenient just before surgery. Several ongoing and published clinical trials are currently evaluating IRDye800‐labeled antibodies targeting alternative biomarkers (VEGFR or EGFR) to distinguish tumor lesions.^34^ However, the retention effect of antibodies due to their large size has been linked with long interval between injection and surgery (several days). In this case, it might be impractical if the surgery is postponed or canceled due to sudden deterioration or any other causes. Peptides have smaller size than antibodies, antibody fragments, and protein scaffolds, which suggests high‐contrast imaging at early timepoints after injection.^35^ In our study, approximately 90% of IRDye800‐RM26 was excreted mainly through the kidney and liver within 24 h. Particularly, approximately 99% of the probe was cleared from the blood at 12 h post injection. The brain with intact and normal functioning blood–brain barrier exhibited low signal all the time. Fast in vivo clearance and less retention in central nervous system contributed to high SBR in a few hours after administration for NIR‐II fluorescence‐guide neurosurgery.

Gross total resection is generally favored for patients with brain malignancies in eloquent area, considering that more aggressive resection tends to minimize the risk of permanent neurological deficits.^36^ Fluorescence guided surgery has been used to maximize tumor resection or control with minimal possible levels of perioperative morbidity. Near‐infrared‐II imaging enhances the tumor to normal tissue ratio,^13^ demonstrating prospect for ameliorating tumor localization and intraoperative surgical margin assessment. In our study, the NIR‐II imaging of IRDye800‐RM26 outperformed that of NIR‐I for visualizing brain tumors. Although this improved performance had been verified,^18^ the feasibility of IRDye800‐labeled fluorescence agents for FGS in NIR‐II window remained unknown. The advantages of NIR‐II imaging guided neurosurgery in mouse models were demonstrated. Specifically, compared to the FDA‐approved dye 5‐ALA, IRDye800‐RM26 had better targeting specificity for glioblastoma. However, the superiority of IRDye800‐RM26 to 5‐ALA regarding clinical diagnostic accuracy needs to be further confirmed.

High in vivo biocompatibility of the probe is the importance of translational advances into clinical practice. To develop a NIR‐II fluorescence agent with a favorable safety profile in humans, IRDye800‐RM26 was used because its components have previously been used in humans without toxicity.^37^, ^38^, ^39^, ^40^, ^41^, ^42^, ^43^ In this study, obvious toxicity was not observed based on blood parameters and H&E stain of vital organs from mice receiving IRDye800‐RM26. The high biocompatibility and well toleration of this probe are appropriate for further clinical translation.

Although surgical interventions are important treatment options for patients with brain malignancies, resection of a cancer specimen during surgery might increase the risk of dissemination and seeding of malignant cells.^44^, ^45^ Surprisingly, we found that inhibited GRPR signaling by low dose (10 μM) IRDye800‐RM26 impeded tumor cell invasion. This observation assumed that the application of this probe might decrease the risk of cancer cell dissemination and relapse brain malignancies after surgery. Nonetheless, this is hypothetical and needs to be proven in vivo.

Overall, the tumor signal and background ratio is an important determinant for the evaluation of the efficacy of a fluorescent agent, can be influenced by selection bias for relevant regions of interest, tumor size, antigen expression, tissue optical properties that are generally variable and unknown in each individual. Although we conducted real‐time NIR‐II imaging in a mouse model through a cranial window to reduce the impact of these factors, the variations of observer measurement and imaging instrument performance still existed. Furthermore, sensitivity and specificity are crucial markers of the diagnostic performance of fluorescence‐guided surgery, and provide insight into the fluorescent agent's targeting efficacy. We performed histological colocalization with Manders' coefficients to prove the fluorescence colocalize with the tumor, and a non‐fluorescent area free of tumor, but further large sample sizes for powered calculation are required to evaluate sensitivity and specificity. Besides, we were not capable of simulating FGS with 5‐ALA for glioblastoma for lack of a surgical imaging system that permit the visualization of 5‐ALA, which limited the comparison between our probe and 5‐ALA in a surgical scenario. A multispectral surgical imaging system needs to be developed that can be equipped with visible and NIR‐I/II available fluorescence options.

## 5 CONCLUSION

In the current study, we developed a new clinically translational‐targeted tracer that could be used to perform NIR‐II fluorescence imaging, which provided the initial proof in preclinical testing of FGS for brain malignancies. It should be noted that the features of imaging and the toxicity of the probe from a preclinical mouse model cannot be deduced directly to humans. Accordingly, the sample size and types of animal models need to be expanded to present a high similarity to human brain tumors. A comprehensive clinical toxicology program is needed to advance clinical translation. Moreover, further studies are needed to develop high‐performance surgical NIR‐II imaging systems that increase sensitivity, contrast, and resolution of fine anatomical structures, and ensure the efficacy and procedural safety during testing in patients.

## AUTHOR CONTRIBUTIONS

**Yuan Zhang:** Data curation (lead); formal analysis (lead); investigation (lead); methodology (equal); project administration (equal); visualization (lead); writing – original draft (lead). **Li Wang:** Resources (equal). **Chengkai Zhang:** Investigation (equal). **Jingjing Zhang:** Conceptualization (equal); resources (equal); writing – review and editing (equal). **Linhao Yuan:** Methodology (equal). **Shucheng Jin:** Methodology (equal). **Wenjianlong Zhou:** Methodology (equal). **Xiudong Guan:** Methodology (equal). **Peng Kang:** Methodology (equal). **Chuanbao Zhang:** Methodology (equal). **Jie Tian:** Conceptualization (equal); methodology (equal); resources (supporting); writing – review and editing (equal). **Xiaoyuan Chen:** Conceptualization (equal); methodology (equal); resources (equal); writing – review and editing (equal). **Deling Li:** Conceptualization (lead); formal analysis (lead); funding acquisition (lead); methodology (equal); project administration (equal); resources (equal); supervision (equal); writing – review and editing (lead). **Wang Jia:** Conceptualization (lead); funding acquisition (lead); investigation (lead); project administration (lead); resources (lead); supervision (lead).

## CONFLICT OF INTEREST STATEMENT

The authors declare no conflicts of interest.

## Supporting information


**Figure S1:** NIR‐II Images of ICG and IRDye800‐RM26 in PBS and FBS during exposure to 808 nm light for 1 h.

**Figure S2:** Images of “tumor” and “peritumor” of glioblastoma for C57BL/6 mice with IRDye800‐RM26 after fluorescence‐guided surgery. (a) NIR‐II fluorescence imaging of “tumor” with high fluorescence intensities (red dotted lines) and “peritumor” with low fluorescence intensities (white dotted lines) of glioblastoma. (b) H&E staining of “tumor” with high fluorescence intensities (HFI) and “peritumor” with low fluorescence intensities (LFI) was conducted. Normal brain tissues with HFI that was verified by H&E staining were shown in red dotted lines, and residual tumor on “peritumor” that was verified by H&E staining were shown in blue dotted lines.

**Figure S3:** Affinity to GRPR. (A) Fluorescence imaging of A549, MDA‐MB231, and GL261 cell lines. GRPR expression was confirmed via ICC/IF (top), IRDye800‐RM26 uptake in all cell lines was high (middle), IRDye800‐RM26 blocking shown reduced IRDye800‐RM26 uptake in all cell lines treated with RM26(bottom). (b) Mean fluorescence intensities for IRDye800‐RM26 uptake (active) and blocking in all cell lines. ★: 0.001 ≤ *p*<0.05, ★★: *p*<0.001.

**Figure S4:** Dynamic NIR‐II fluorescence imaging of C57BL/6 mice organs representing the biodistribution and excretion of 40 μg IRDye800‐RM26.

**Figure S5:** Histology (H&E staining) of organs (3 days post injection of 40 μg IRDye800‐RM26).

**Figure S6:** Effects of IRDy800‐RM26 on growth and invasion in cell lines. (a) In vitro growth of A549 cell lines after addition different concentrations of IRDye800‐RM26 for 24 h. (b) In vitro growth of A549, MDA‐MB231 and GL261 cell lines after addition 10 μM IRDye800‐RM26. (c and d) Images and quantification of invasion in different concentrations of IRDye800‐RM26 treated cell lines for 24 h. ★: 0.001 ≤ *p*<0.05, ★★: *p*<0.001.

**Table S1.** Univariate analyses of the blood routine test results of C57/BL6 mice (aged 6 months) before and at 3 days after intravenous administration.

**Table S2.** Univariate analyses of the blood biochemistry test results of C57/BL6 mice (aged 6 months) before and at 3, 7, and 14 days after intravenous administration.

**Data S1:** Supporting information

## Data Availability

The data that support the findings of this study are available from the corresponding author upon reasonable request.
