# Supplementary figures and images for: Preclinical assessment of IRDye800CW‐labeled gastrin‐releasing peptide receptor‐targeting peptide for near infrared‐II imaging of brain malignancies
Source: Bioeng Transl Med. 2023 May 9;8(4):e10532. doi: 10.1002/btm2.10532 (PMC10354759; doi:10.1002/btm2.10532)

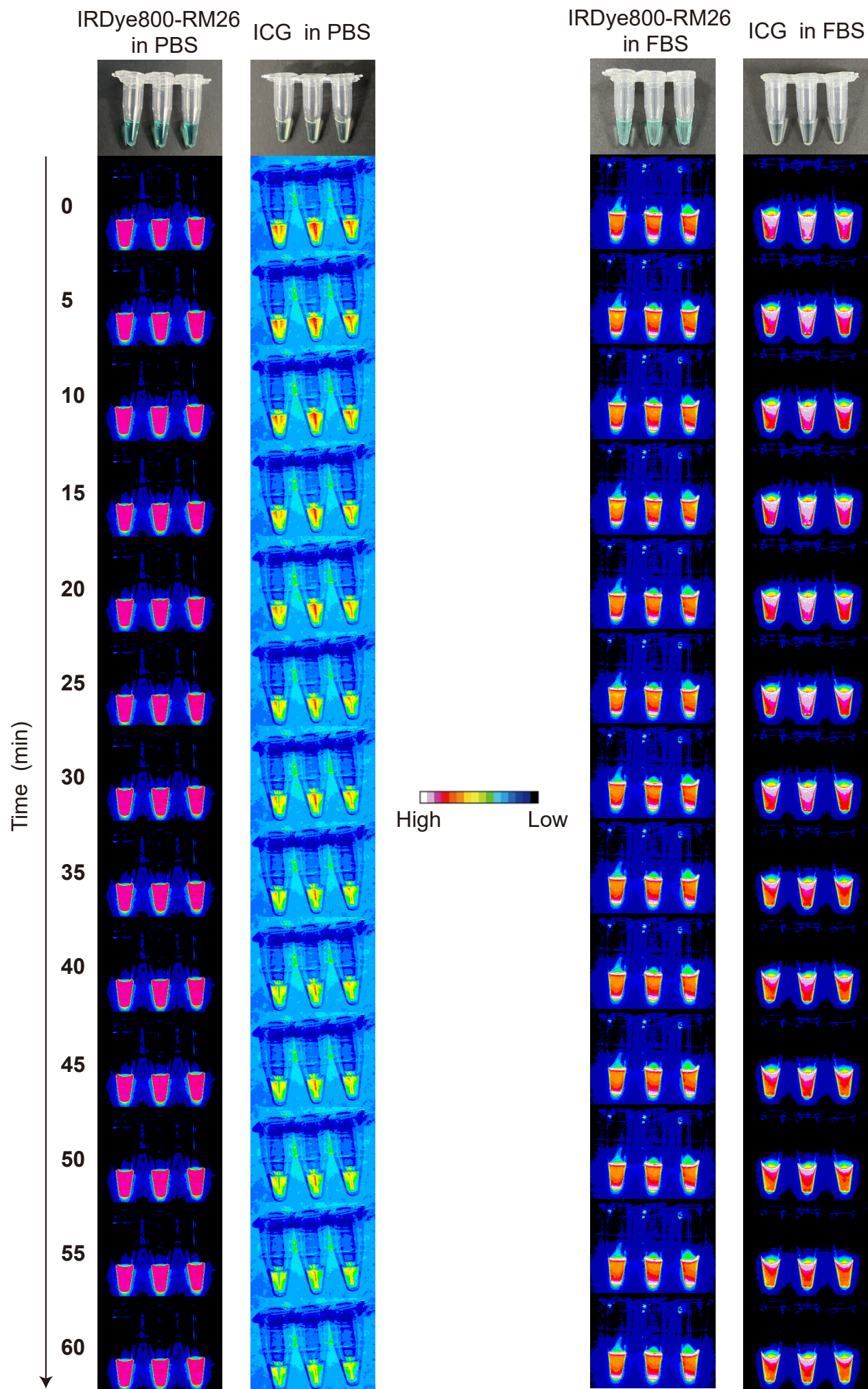

Supplement: Supplementary file 1 — Figure S1: NIR‐II Images of ICG and IRDye800‐RM26 in PBS and FBS during exposure to 808 nm light for 1 h. [file BTM2-8-e10532-s002.pdf]

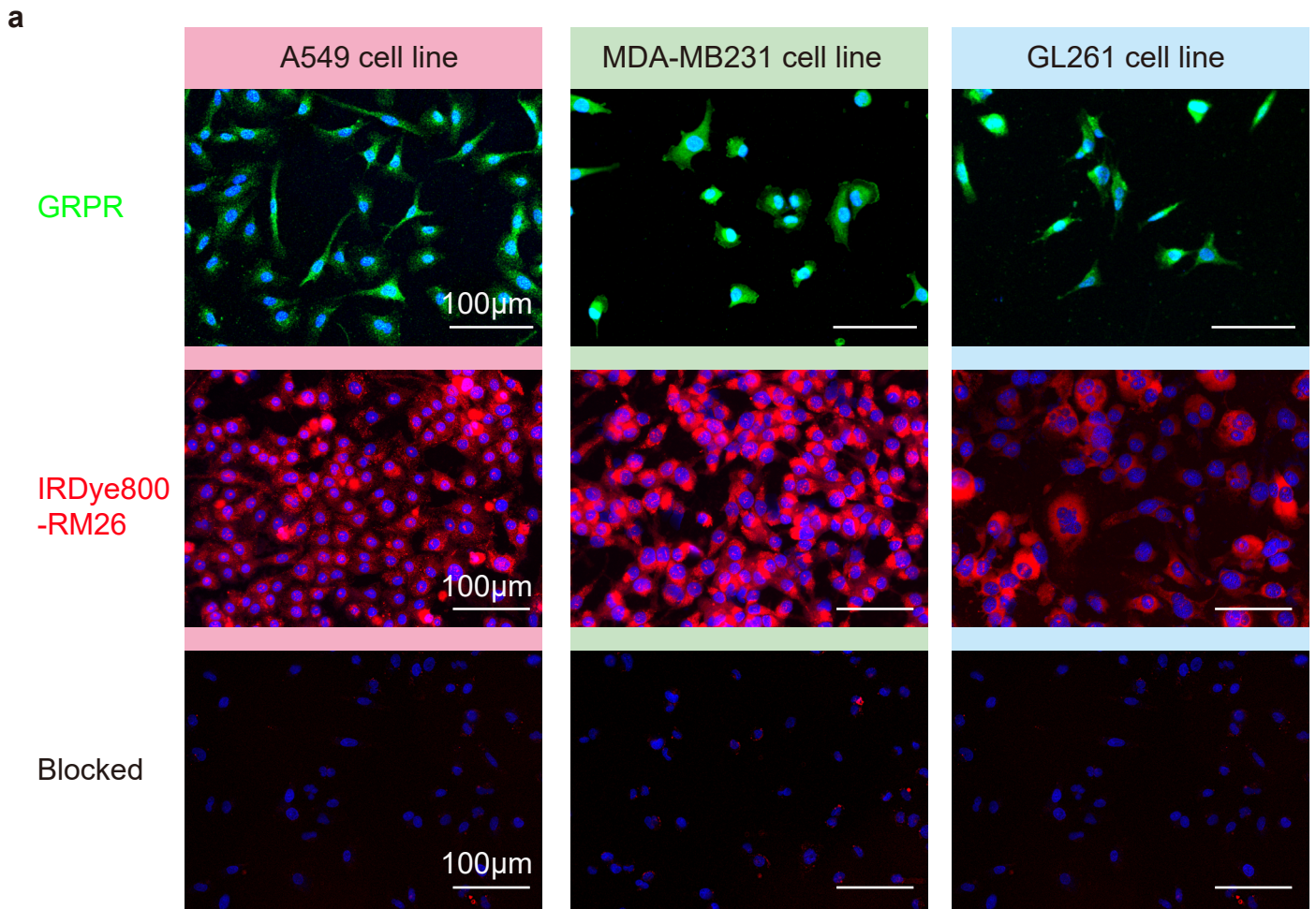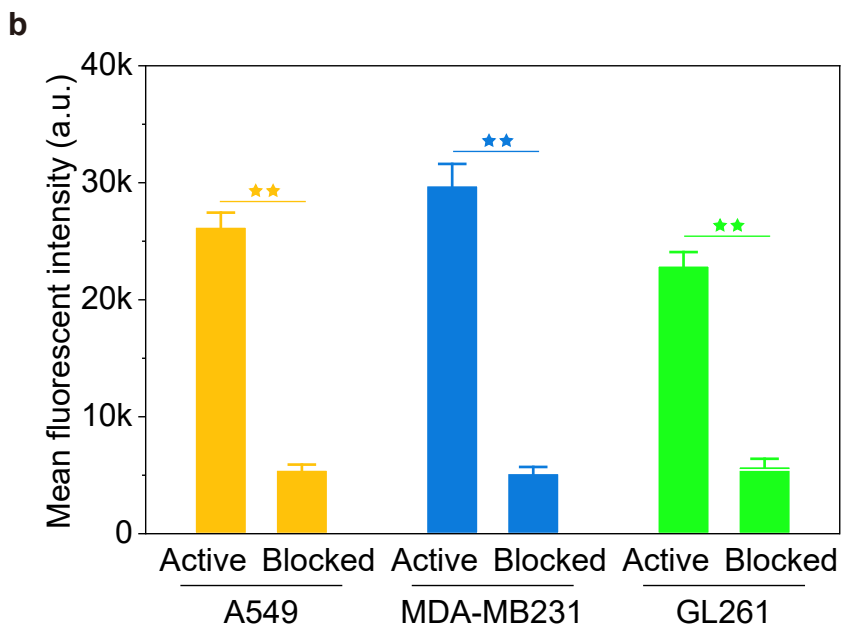

Supplement: Supplementary file 3 — Figure S3: Affinity to GRPR. (A) Fluorescence imaging of A549, MDA‐MB231, and GL261 cell lines. GRPR expression was confirmed via ICC/IF (top), IRDye800‐RM26 uptake in all cell lines was high (middle), IRDye800‐RM26 blocking shown reduced IRDye800‐RM26 uptake in all cell lines treated with RM26(bottom). (b) Mean fluorescence intensities for IRDye800‐RM26 uptake (active) and blocking in all cell lines. ★: 0.001 ≤ p<0.05, ★★: p<0.001. [file BTM2-8-e10532-s008.pdf]

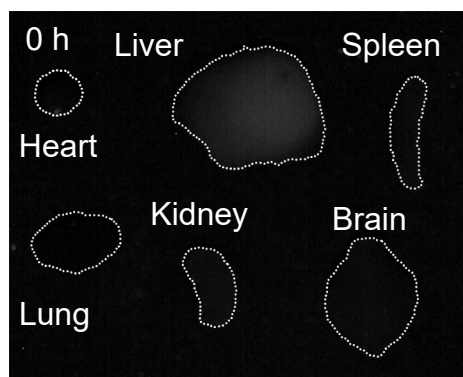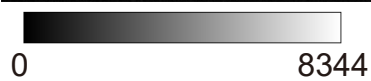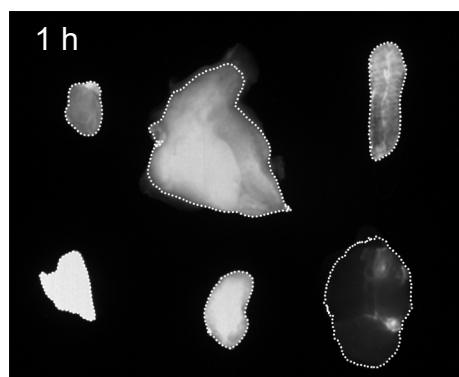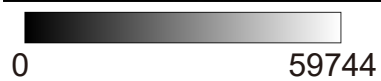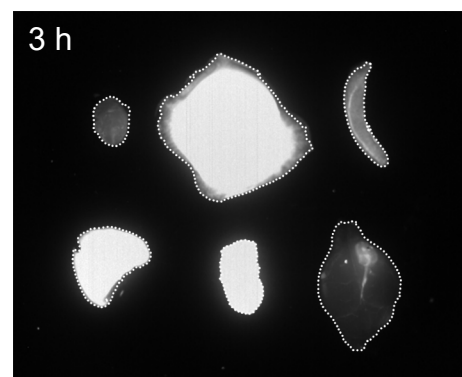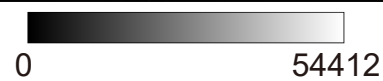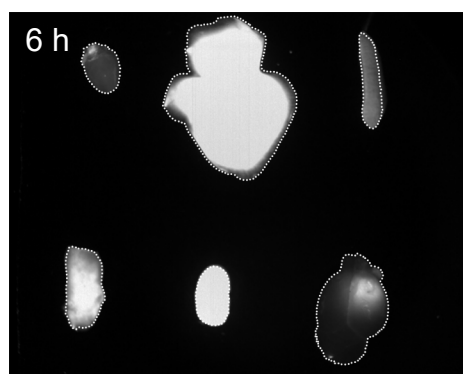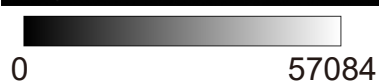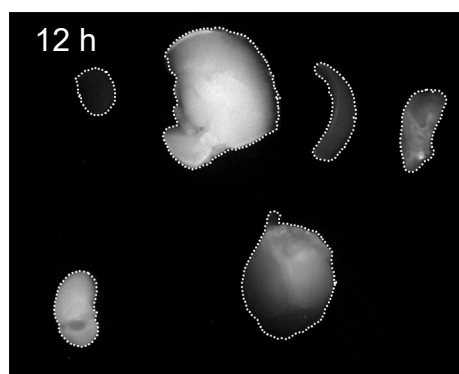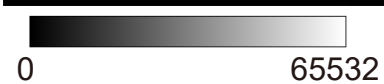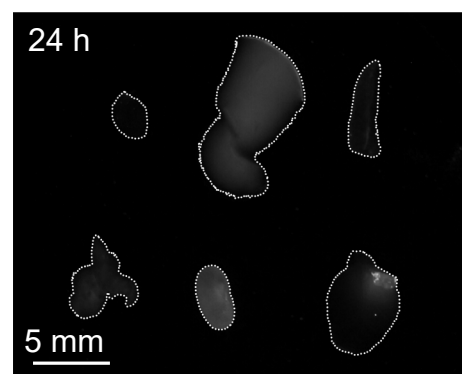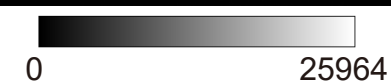

Supplement: Supplementary file 4 — Figure S4: Dynamic NIR‐II fluorescence imaging of C57BL/6 mice organs representing the biodistribution and excretion of 40 μg IRDye800‐RM26. [file BTM2-8-e10532-s006.pdf]

IRDye800-RM26 (40  $\mu$ g)

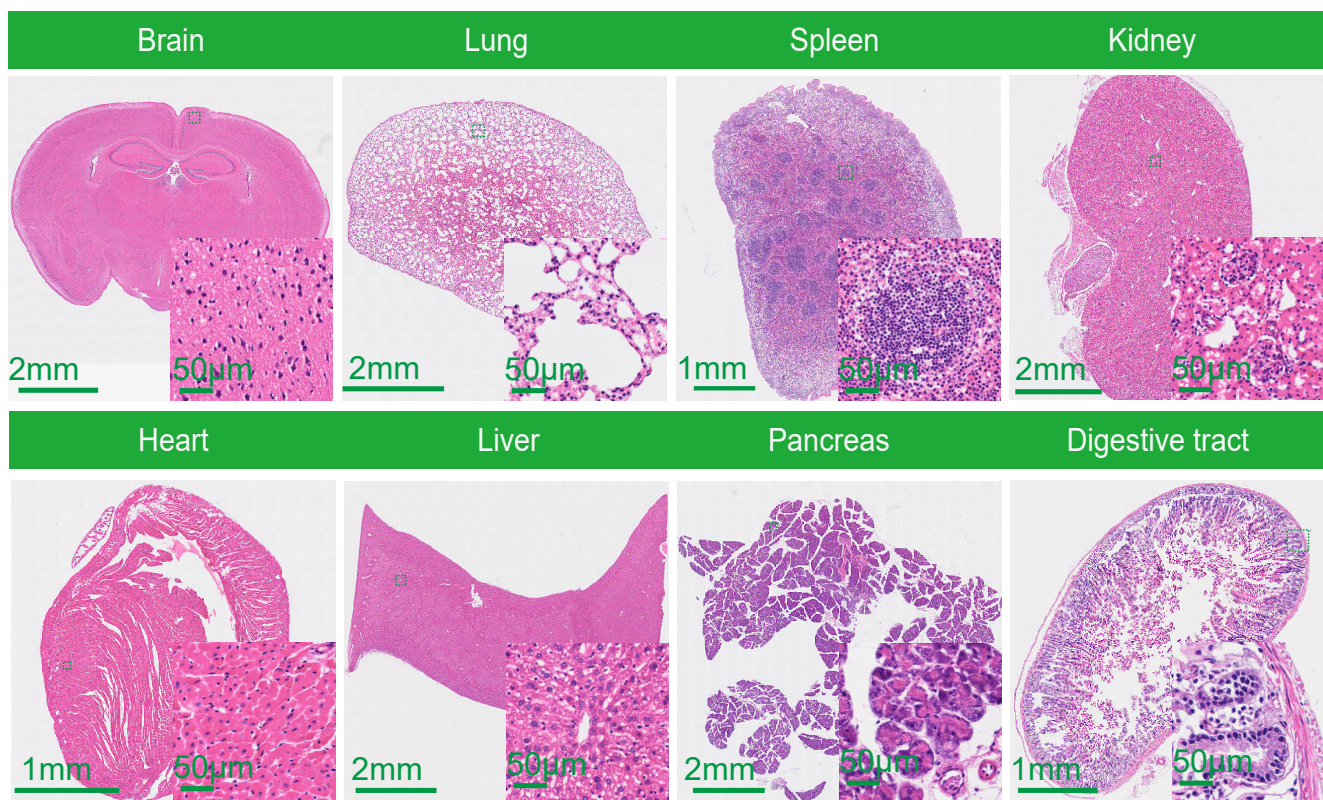

Equal volume PBS

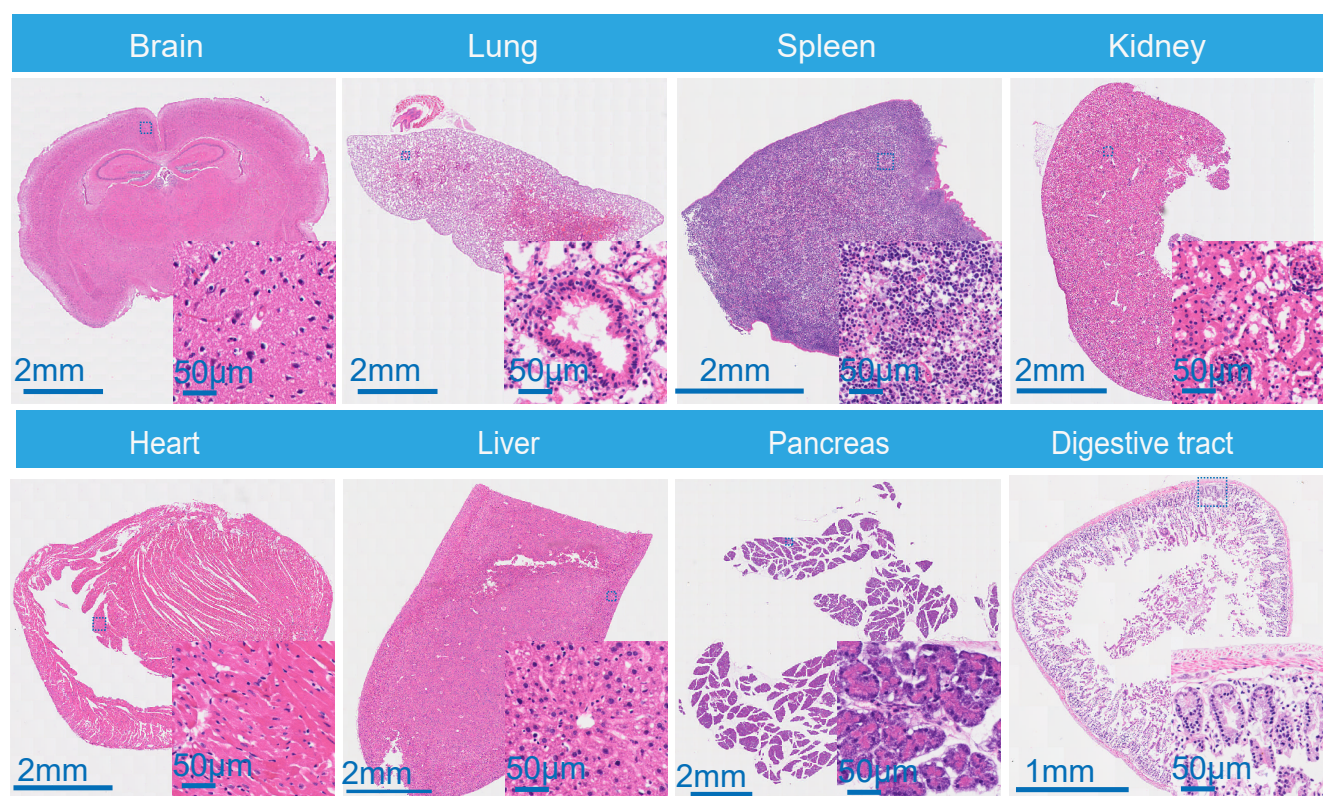

Supplement: Supplementary file 5 — Figure S5: Histology (H&E staining) of organs (3 days post injection of 40 μg IRDye800‐RM26). [file BTM2-8-e10532-s001.pdf]

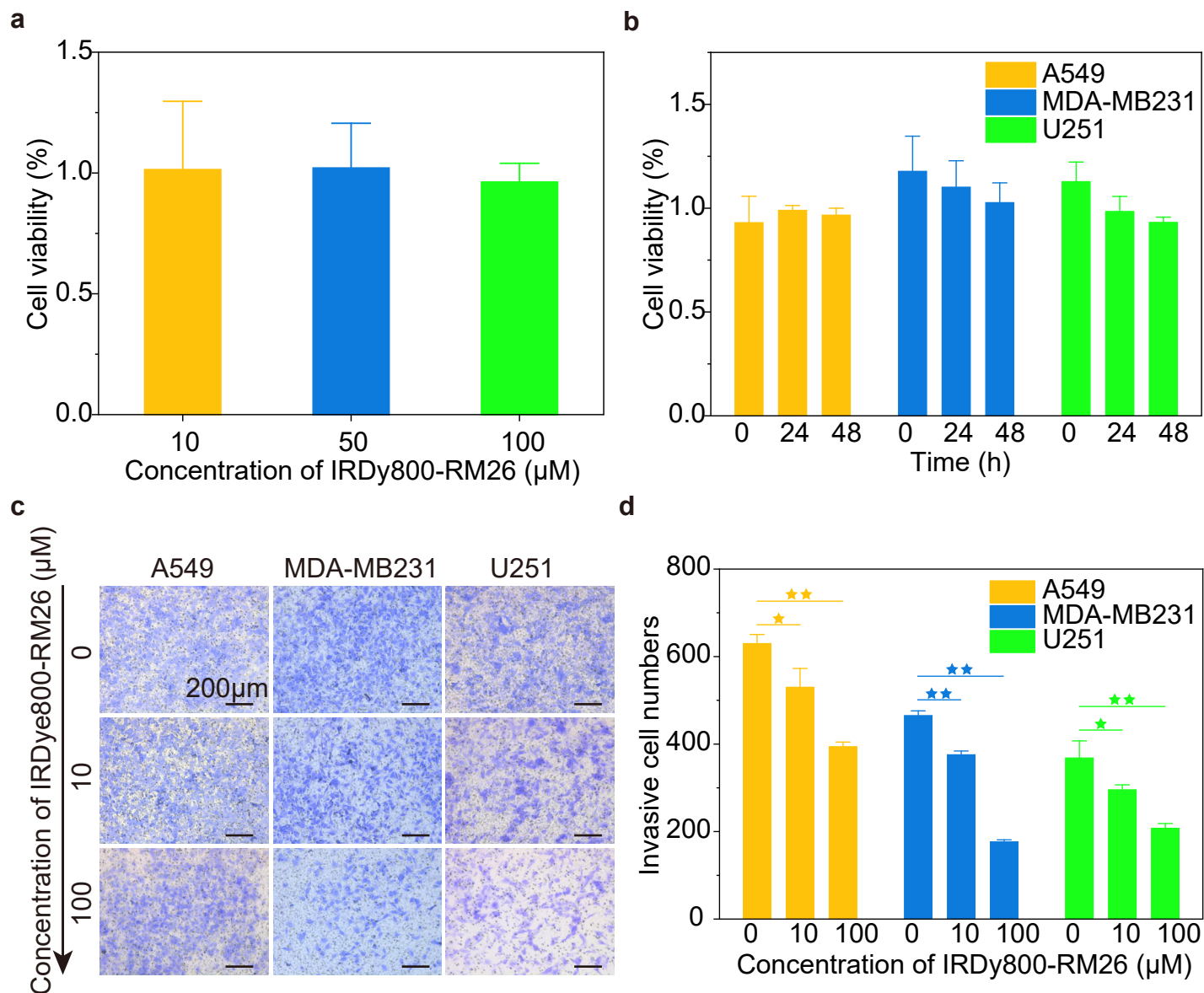

Supplement: Supplementary file 6 — Figure S6: Effects of IRDy800‐RM26 on growth and invasion in cell lines. (a) In vitro growth of A549 cell lines after addition different concentrations of IRDye800‐RM26 for 24 h. (b) In vitro growth of A549, MDA‐MB231 and GL261 cell lines after addition 10 μM IRDye800‐RM26. (c and d) Images and quantification of invasion in different concentrations of IRDye800‐RM26 treated cell lines for 24 h. ★: 0.001 ≤ p<0.05, ★★: p<0.001. [file BTM2-8-e10532-s004.pdf]
