# Supplementary material for: Preclinical assessment of IRDye800CW‐labeled gastrin‐releasing peptide receptor‐targeting peptide for near infrared‐II imaging of brain malignancies
Source: Bioeng Transl Med. 2023 May 9;8(4):e10532. doi: 10.1002/btm2.10532 (PMC10354759; doi:10.1002/btm2.10532)
Supplement: Supplementary file 7 — Table S1. Univariate analyses of the blood routine test results of C57/BL6 mice (aged 6 months) before and at 3 days after intravenous administration. [file BTM2-8-e10532-s007.pdf]

Supplementary Table S1. Univariate analyses of the blood routine test results of C57/BL6 mice (aged 6 months) before and at 3 days after intravenous administration.

	Before	Day 3	Reference §	P
WBC (10^9/L)	1.680±0.325	2.450±0.416	2.20-11.53	0.065
RBC (10^12/L)	6.377±0.922	7.203±1.234	3.47-11.73	0.405
HGB (g/L)	97.667±14.012	110.000±17.776	57.000-170.000	0.399
HCT (%)	44.700±5.738	51.133±8.923	16.200-58.300	0.353
MCV (fL)	70.233±1.266	70.967±0.321	-	0.386
MCH (pg)	15.300±0.265	15.267±0.153	-	0.859
MCHC (g/L)	218.333±5.774	215.333±2.887	-	0.466
PLT (10^9/L)	58.000±45.902	162.000±63.930	144.00-894.00	0.084
RDW-SD (fL)	33.233±0.462	34.167±1.193	-	0.275
RDW-CV (%)	13.400±0.265	14.400±1.732	-	0.379
PDW (fL)	6.433±0.153	7.200±0.361	-	0.028
MPV (fL)	7.300±0.200	7.800±0.173	-	0.031
P-LCR (%)	4.967±1.721	7.933±1.550	-	0.091
PCT (%)	0.040±0.036	0.127±0.047	-	0.065
NEUT# (10^9/L)	0.043±0.029	0.083±0.015	-	0.101
LYMPH# (10^9/L)	1.543±0.301	2.013±0.345	-	0.150
MONO# (10^9/L)	0.033±0.006	0.210±0.226	-	0.309
EO# (10^9/L)	0.000±0.000	0.000±0.000	-	-
BASO# (10^9/L)	0.060±0.000	0.143±0.076	-	0.199
NEUT% (%)	2.467±1.474	3.433±0.115	-	0.374
LYMPH% (%)	91.833±1.242	82.567±10.318	-	0.197
MONO% (%)	2.033±0.473	8.100±7.674	-	0.244
EO% (%)	0.000±0.000	0.000±0.000	-	-
BASO% (%)	3.667±0.702	5.900±3.041	-	0.283

§2.5th–97.5th percentiles interval of hematological parameters from study by Cristina Mazzaccara, et al (doi: 10.1371/journal.pone.0003772).
