# Supplementary material for: Preclinical assessment of IRDye800CW‐labeled gastrin‐releasing peptide receptor‐targeting peptide for near infrared‐II imaging of brain malignancies
Source: Bioeng Transl Med. 2023 May 9;8(4):e10532. doi: 10.1002/btm2.10532 (PMC10354759; doi:10.1002/btm2.10532)
Supplement: Supplementary file 8 — Table S2. Univariate analyses of the blood biochemistry test results of C57/BL6 mice (aged 6 months) before and at 3, 7, and 14 days after intravenous administration. [file BTM2-8-e10532-s005.pdf]

Supplementary Table S2. Univariate analyses of the blood biochemistry test results of C57/BL6 mice (aged 6 months) before and at 3, 7, and 14 days after intravenous administration.

	Before	Day 3	Day 7	Day 14	Reference [§]	P
TP (g/L)	59.261±0.272	60.632±1.372	58.847±0.559	58.336±3.260	45-83	0.482
AST (U/L)	82.535±0.480	117.445±11.518	89.750±13.149	94.317±12.855	51-122	0.021
ALT (U/L)	38.871±0.779	47.885±6.044	35.479±4.815	32.719±2.744	42-73	0.010
TC (mmol/L)	2.328±0.018	2.512±0.110	2.167±0.148	2.356±0.177	1.3-3.4	0.065
CREA (μmol/L)	36.784±1.381	39.925±3.319	32.591±2.997	31.803±2.317	-	0.017
UREA (mmol/L)	12.706±0.277	11.165±0.477	8.325±0.999	9.828±0.384	-	0.000
GLU (mmol/L)	8.893±0.008	8.173±0.378	7.683±0.637	7.486±0.467	5.2-12.2	0.018
TBIL (μmol/L)	2.517±0.386	6.830±1.308	3.293±1.879	0.789±0.489	3.4-14.3	0.002
ALP (U/L)	250.063±0.434	152.560±46.364	224.058±14.096	239.719±12.341	103-217	0.006
Ca (mmol/L)	2.573±0.274	2.029±0.025	2.136±0.128	2.285±0.042	2.3-3.5	0.012
Na (mmol/L)	142.181±0.754	145.813±8.785	148.744±9.710	153.143±4.005	149.0-281.4	0.319
K (mmol/L)	8.453±0.045	8.674±0.465	5.202±0.061	6.264±0.044	4.0-14.0	0.000

§2.5th–97.5th percentiles interval of hematological parameters from study by Cristina Mazzaccara, et al (doi: 10.1371/journal.pone.0003772).
