# Supplementary material for: Preclinical assessment of IRDye800CW‐labeled gastrin‐releasing peptide receptor‐targeting peptide for near infrared‐II imaging of brain malignancies
Source: Bioeng Transl Med. 2023 May 9;8(4):e10532. doi: 10.1002/btm2.10532 (PMC10354759; doi:10.1002/btm2.10532)
Supplement: Supplementary file 9 — Data S1: Supporting information [file BTM2-8-e10532-s009.docx]

**Supplementary figure legends:**

**Supplementary Fig. S1:** NIR-II Images of ICG and IRDye800-RM26 in PBS and FBS during exposure to 808 nm light for 1 h**.**

**Supplementary Fig. S2:** Images of “tumor” and “peritumor” of glioblastoma for C57BL/6 mice with IRDye800-RM26 after fluorescence-guided surgery. a) NIR-II fluorescence imaging of “tumor” with high fluorescence intensities (red dotted lines) and “peritumor” with low fluorescence intensities (white dotted lines) of glioblastoma. b) H&E staining of “tumor” with high fluorescence intensities (HFI) and “peritumor” with low fluorescence intensities (LFI) was conducted. Normal brain tissues with HFI that was verified by H&E staining were shown in red dotted lines, and residual tumor on “peritumor” that was verified by H&E staining were shown in blue dotted lines.

**Supplementary Fig. S3:** Affinity to GRPR. A) Fluorescence imaging of A549, MDA-MB231, and GL261 cell lines. GRPR expression was confirmed via ICC/IF (top), IRDye800-RM26 uptake in all cell lines was high (middle), IRDye800-RM26 blocking shown reduced IRDye800-RM26 uptake in all cell lines treated with RM26(bottom). b) Mean fluorescence intensities for IRDye800-RM26 uptake (active) and blocking in all cell lines. 🟊: 0.001≤p＜0.05, 🟊🟊: p＜0.001.

**Supplementary Fig. S4:** Dynamic NIR-II fluorescence imaging of C57BL/6 mice organs representing the biodistribution and excretion of 40 μg IRDye800-RM26.

**Supplementary Fig. S5:** Histology (H&E staining) of organs (3 days post injection of 40 μg IRDye800-RM26).

**Supplementary Fig. S6:** Effects of IRDy800-RM26 on growth and invasion in cell lines. a) In vitro growth of A549 cell lines after addition different concentrations of IRDye800-RM26 for 24 h. b) In vitro growth of A549, MDA-MB231 and GL261 cell lines after addition 10 μM IRDye800-RM26. c, d) Images and quantification of invasion in different concentrations of IRDye800-RM26 treated cell lines for 24 h. 🟊: 0.001≤p＜0.05, 🟊🟊: p＜0.001.

**Supplementary tables**

| **Supplementary Table S1.** Univariate analyses of the blood routine test results of C57/BL6 mice (aged 6 months) before and at 3 days after intravenous administration. | | | | |
| --- | --- | --- | --- | --- |
|  | Before | Day 3 | Reference ^§^ | P |
| WBC (10^9/L) | 1.680±0.325 | 2.450±0.416 | 2.20-11.53 | 0.065 |
| RBC (10^12/L) | 6.377±0.922 | 7.203±1.234 | 3.47-11.73 | 0.405 |
| HGB (g/L) | 97.667±14.012 | 110.000±17.776 | 57.000-170.000 | 0.399 |
| HCT (%) | 44.700±5.738 | 51.133±8.923 | 16.200-58.300 | 0.353 |
| MCV (fL) | 70.233±1.266 | 70.967±0.321 | - | 0.386 |
| MCH (pg) | 15.300±0.265 | 15.267±0.153 | - | 0.859 |
| MCHC (g/L) | 218.333±5.774 | 215.333±2.887 | - | 0.466 |
| PLT (10^9/L) | 58.000±45.902 | 162.000±63.930 | 144.00-894.00 | 0.084 |
| RDW-SD (fL) | 33.233±0.462 | 34.167±1.193 | - | 0.275 |
| RDW-CV (%) | 13.400±0.265 | 14.400±1.732 | - | 0.379 |
| PDW (fL) | 6.433±0.153 | 7.200±0.361 | - | 0.028 |
| MPV (fL) | 7.300±0.200 | 7.800±0.173 | - | 0.031 |
| P-LCR (%) | 4.967±1.721 | 7.933±1.550 | - | 0.091 |
| PCT (%) | 0.040±0.036 | 0.127±0.047 | - | 0.065 |
| NEUT# (10^9/L) | 0.043±0.029 | 0.083±0.015 | - | 0.101 |
| LYMPH# (10^9/L) | 1.543±0.301 | 2.013±0.345 | - | 0.150 |
| MONO# (10^9/L) | 0.033±0.006 | 0.210±0.226 | - | 0.309 |
| EO# (10^9/L) | 0.000±0.000 | 0.000±0.000 | - | - |
| BASO# (10^9/L) | 0.060±0.000 | 0.143±0.076 | - | 0.199 |
| NEUT% (%) | 2.467±1.474 | 3.433±0.115 | - | 0.374 |
| LYMPH% (%) | 91.833±1.242 | 82.567±10.318 | - | 0.197 |
| MONO% (%) | 2.033±0.473 | 8.100±7.674 | - | 0.244 |
| EO% (%) | 0.000±0.000 | 0.000±0.000 | - | - |
| BASO% (%) | 3.667±0.702 | 5.900±3.041 | - | 0.283 |

^§^2.5th–97.5th percentiles interval of hematological parameters from study by Cristina Mazzaccara, et al (1).

| **Supplementary Table S2.** Univariate analyses of the blood biochemistry test results of C57/BL6 mice (aged 6 months) before and at 3, 7, and 14 days after intravenous administration. | | | | | | |
| --- | --- | --- | --- | --- | --- | --- |
|  | Before | Day 3 | Day 7 | Day 14 | Reference ^§^ | P |
| TP (g/L) | 59.261±0.272 | 60.632±1.372 | 58.847±0.559 | 58.336±3.260 | 45-83 | 0.482 |
| AST (U/L) | 82.535±0.480 | 117.445±11.518 | 89.750±13.149 | 94.317±12.855 | 51-122 | 0.021 |
| ALT (U/L) | 38.871±0.779 | 47.885±6.044 | 35.479±4.815 | 32.719±2.744 | 42-73 | 0.010 |
| TC (mmol/L) | 2.328±0.018 | 2.512±0.110 | 2.167±0.148 | 2.356±0.177 | 1.3-3.4 | 0.065 |
| CREA (μmol/L) | 36.784±1.381 | 39.925±3.319 | 32.591±2.997 | 31.803±2.317 | - | 0.017 |
| UREA (mmol/L) | 12.706±0.277 | 11.165±0.477 | 8.325±0.999 | 9.828±0.384 | - | 0.000 |
| GLU (mmol/L) | 8.893±0.008 | 8.173±0.378 | 7.683±0.637 | 7.486±0.467 | 5.2-12.2 | 0.018 |
| TBIL (μmol/L) | 2.517±0.386 | 6.830±1.308 | 3.293±1.879 | 0.789±0.489 | 3.4-14.3 | 0.002 |
| ALP (U/L) | 250.063±0.434 | 152.560±46.364 | 224.058±14.096 | 239.719±12.341 | 103-217 | 0.006 |
| Ca (mmol/L) | 2.573±0.274 | 2.029±0.025 | 2.136±0.128 | 2.285±0.042 | 2.3-3.5 | 0.012 |
| Na (mmol/L) | 142.181±0.754 | 145.813±8.785 | 148.744±9.710 | 153.143±4.005 | 149.0-281.4 | 0.319 |
| K (mmol/L) | 8.453±0.045 | 8.674±0.465 | 5.202±0.061 | 6.264±0.044 | 4.0-14.0 | 0.000 |

^§^2.5th–97.5th percentiles interval of serum biochemical parameters from study by Cristina Mazzaccara, et al (1).

**Supplementary references**

1. Mazzaccara C, Labruna G, Cito G, Scarfo M, De Felice M, Pastore L, Sacchetti L. Age-related reference intervals of the main biochemical and hematological parameters in C57BL/6J, 129SV/EV and C3H/HeJ mouse strains. PLoS One **2008**;3(11):e3772. doi: 10.1371/journal.pone.0003772.

**Supplementary Materials and methods**

**Absorption and Emission**

The IRDye800-RM26 emission profiles were detected in phosphate buffer saline (PBS) or fetal bovine serum (FBS) with FLS980 Series of Fluorescence Spectrometers (Edinburgh Instruments, UK). The emission profile was measured at λ_excitation_ = 758 nm. Absorption was measured on a UV-3600 Plus UV-VIS-NIR spectrophotometer (Shimadzu, Japan).

**The concentration-dependent fluorescence intensity**

The concentration-dependent fluorescence intensity was evaluated by ten different concentrations (0 μM, 0.0001 μM, 0.001 μM, 0.01 μM, 0.1 μM, 1 μM, 10 μM, 100 μM, 1000 μM, and powder) of IRDye800-RM26 in 1×FBS placed in 0.2 ml thin wall PCR tubes (Axygen, US). The tubes were excited by an 808 nm laser at 500 ms exposure time, and illuminated with approximately 1.3 mW/cm^2^ of light. The mean fluorescent intensity (MFI) was calculated from region of interests within the tubes and normalized to the 100 μM.

**Photostability and brightness of IRDye800-RM26 compared with Indocyanine green (ICG)**

100 μM IRDye800-RM26 and 0.01 mg/ml ICG were dissolved in PBS and FBS. The exposure time were 500 ms in PBS, and those were 100ms in FBS. The probes were excited by an 808 nm laser and illuminated with approximately 1.3 mW/cm^2^ of light for 1 h. The mean fluorescence intensities of IRDye800-RM26 and ICG were calculated from region of interests.

**Cell lines culturing**

A549, MDA-MB231 (National Infrastructure of Cell Line Resource, Beijing, China) and GL261, U251 (gift of Dr. Li, Capital Medical University) were cultured in Dulbecco’s Modified Eagle’s medium (Gibco, Carlsbad, CA) added 10% fetal bovine serum (Gibco, Carlsbad, CA) and 1 % Penicillin-Streptomycin (Gibco, Carlsbad, CA) at 37 °C in humid 5 % CO_2_ air. GL261 cells stably expressed firefly luciferase. The cells were passaged or harvested when reaching 80 – 90 % confluency.

**Expression of GRPR in cell lines**

The expression of GRPR in cell lines were qualitatively analyzed by immunofluorescence staining. Briefly, A549, MDA-MB231 and GL261 cells were cultured on sterile glass cover slips overnight at 37 ℃, and then fixed in 4% paraformaldehyde (Biosharp, China) for 20 min. After being washed in 1×PBS 3 times for 5 min by shaker each time, the cells were blocked with 10 % goat serum (ZSGB-BIO, China) for 1 hour, then incubated with GRPR antibody (NLS831; NOVUS, USA) at 1:500 dilution in 10 % goat serum overnight at 4 ℃. The cells were washed in PBS-Tween20 (T8220, Solarbio, China) 3 times for 5 min and incubated with Alexa Fluor® 488 (ab150077; Abcam, UK) at 1:1000 dilution in 10 % goat serum for 30 min at room temperature. Afterwards, the cells were washed in PBS-Tween20 3 times for 5 min again and incubated with DAPI (C0065, Solarbio, China) for 5 min. Then, the cells were washed 3 times with PBS-Tween20 (5 min for each time) and visualize by a confocal laser scanning microscope (LSM 710; ZEISS, Germany).

**Animal models**

6 weeks old female Balb/c nude mice and C57BL/6 mice (Vital River Experimental Animal Technology Co. Beijing, China) were respectively xenografted with A549 and GL261 cells. Briefly, the animals were anesthetized with 2,2,2-Tribromoethanol (Sigma-Aldrich, USA) + 2-Methyl-2-butanol (Sangon Biotech, Shanghai, China) + normal saline in the ratio 1 g: 5 ml: 45 ml by subcutaneous injection of 240 mg/kg body weight of the solution. The tumor model was established by inoculation of 400,000 A549 and GL261 cells in 4 μl ice cold PBS in the right hemisphere (1 mm lateral and 1 mm posterior to the bregma at 2 mm depth) using a stereotaxic frame (RWD, Shenzhen, China) with the automated microinjection pump (RWD, Shenzhen, China). The cells were injected with a flow rate of 1 μl/min with the syringe kept in place for further 2 min before retraction. Tumor growth was subsequently monitored on MRI with the BioclinScan 7 T (Bruker, Germany) with axial T2-weighted and enhanced sequences or IVIS Spectrum In Vivo Imaging System (PerkinElmer, USA). When the tumor formations were detected, the animals were used for the fluorescence imaging experiments.

The cranial window implantation was as the following procedures. Tumor-bearing mice were anesthetized with 2,2,2-Tribromoethanol. Surgery was done under aseptic conditions. The head of mouse was fixed by a stereotactic apparatus (RWD, Shenzhen, China). The skin on top of the frontal and parietal regions of the skull was cleaned with antimicrobial betadine solution. A longitudinal incision of the skin was made from forehead to lambdoidal suture. The skin was then cut in a circular manner on top of the skull, and the periosteum underneath was scraped off to the temporal crests. A 4 mm circle was drawn over the frontal and parietal regions of the skull bilaterally. Using a hand-held cranial drill (RWD, Shenzhen, China) with a burr tip size of 0.5 mm in diameter, a groove was made on the margin of the drawn circle. This groove was made thinner by cautious and continuous drilling of the groove till the bone flap became loose. Cold saline was applied during the drilling process to avoid thermal injury of the cortical regions. Using a blunt microblade, the bone flap was separated from the dura mater underneath. After removal of the bone flap, the dura mater was continuously kept moist with physiological saline. After removal of the bone, the window was then sealed with a 5 mm cover glass by adhering to the bone using a histocompatible cyanoacrylate glue and polymethyl methacrylate that is the most frequently used denture base material in clinic. The surgery took 45 min in each case and was followed by positioning of the animals on a heating pad (37℃) until they recovered from the anesthesia.

**Proliferation and invasion assays**

To evaluate potential inhibition of IRDye800-RM26 on tumor cell proliferation and invasion, standard Cell Counting Kit-8 (CCK8) and Transwell assays on lung cancer cell A549, breast cancer cell MDA-MB231, and glioblastoma cell U251 were performed.

The proliferation assay of IRDye800-RM26 was examined using a standard CCK method (Dojindo, USA). 100 μl of A549 cells suspension (5000 cells/well) were seeded in 96-well plates and incubated the plate overnight (37°C, 5% CO_2_). The cells were incubated with different concentrations of IRDye800-RM26 (10 μM, 50 μM and 100 μM) for 24 h. Then 10 μl of CCK-8 solution to each well of the plate, and incubated the plate for 2 h in the incubator (37°C, 5% CO_2_). The relative cell viability (%) was measured with a microplate reader (Spark, TECAN, Switzerland) at 450 nm absorbance. The cell viabilities of A549, MDA-MB231 and U251 cells were also measured after incubated with 10 μM IRDye800-RM26 for 24 h. Three independent experiments were performed.

For invasion assay, the transwell filters were coated with 1/30 diluted Matrigel (Corning, USA). A549, MDA-MB231 and U251 cells (1 × 10^5^) in serum-free media were added to the upper chamber and 10 % serum media was added to the lower chamber. After 24 h incubation with different concentrations of IRDye800-RM26 (10 μM, 50 μM and 100 μM), cells were fixed with 4% paraformaldehyde, stained with 0.1% crystal violet ammonium oxalate solution (Solarbio, China), and counted.
