# Supplementary material for: Preclinical assessment of IRDye800CW‐labeled gastrin‐releasing peptide receptor‐targeting peptide for near infrared‐II imaging of brain malignancies
Source: Bioeng Transl Med. 2023 May 9;8(4):e10532. doi: 10.1002/btm2.10532 (PMC10354759; doi:10.1002/btm2.10532)
Supplement: Supplementary file 2 — Figure S2: Images of “tumor” and “peritumor” of glioblastoma for C57BL/6 mice with IRDye800‐RM26 after fluorescence‐guided surgery. (a) NIR‐II fluorescence imaging of “tumor” with high fluorescence intensities (red dotted lines) and “peritumor” with low fluorescence intensities (white dotted lines) of glioblastoma. (b) H&E staining of “tumor” with high fluorescence intensities (HFI) and “peritumor” with low fluorescence intensities (LFI) was conducted. Normal brain tissues with HFI that was verified by H&E staining were shown in red dotted lines, and residual tumor on “peritumor” that was verified by H&E staining were shown in blue dotted lines. [file BTM2-8-e10532-s003.pdf]

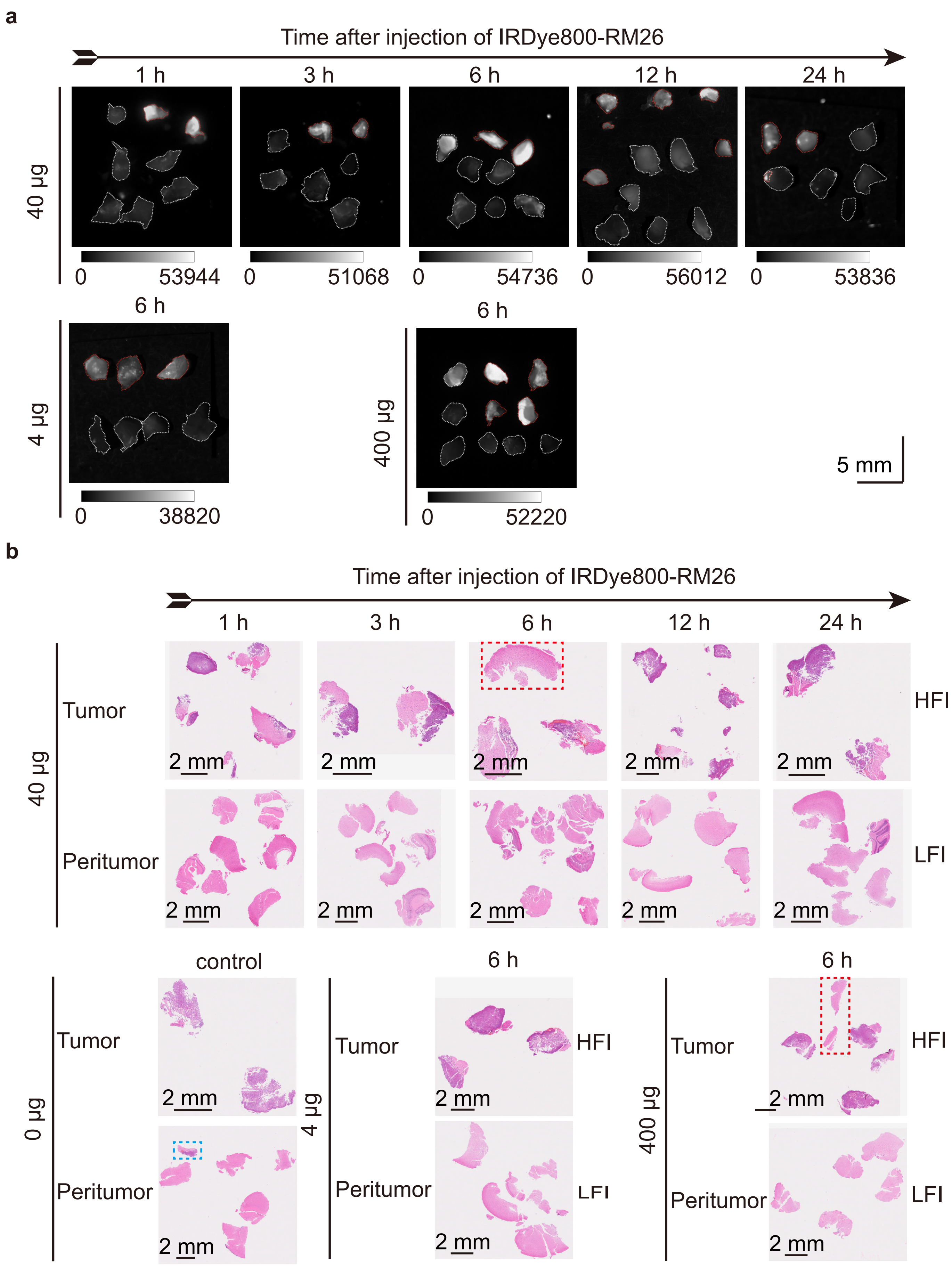
a
Time after injection of IRDye800-RM26
1 h
3 h
6 h
12 h
24 h
40 μg
0
53944
0
51068
0
54736
0
56012
0
53836
6 h
4 μg
0
38820
6 h
400 μg
0
52220
5 mm
b
Time after injection of IRDye800-RM26
1 h
3 h
6 h
12 h
24 h
40 μg
Tumor
Peritumor
2 mm
HFI
LFI
control
0 μg
6 h
4 μg
6 h
400 μg
